# Supplementary figures and images for: Extracellular matrix analysis of fibrosis: A step towards tissue engineering for urethral stricture disease
Source: PLoS One. 2023 Nov 30;18(11):e0294955. doi: 10.1371/journal.pone.0294955 (PMC10688748; doi:10.1371/journal.pone.0294955)

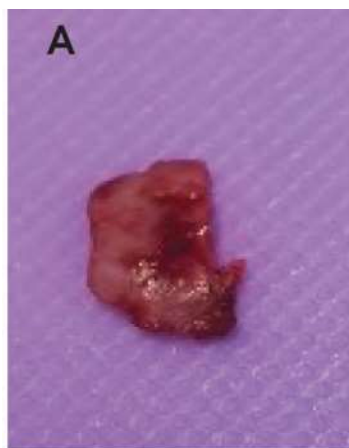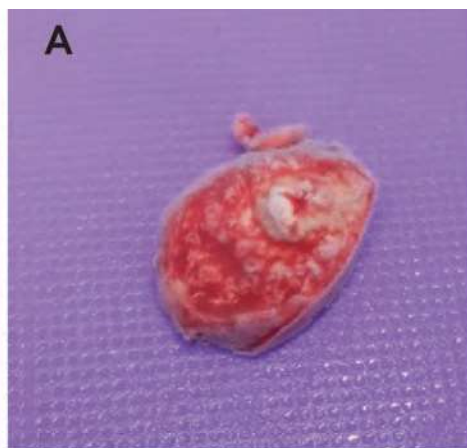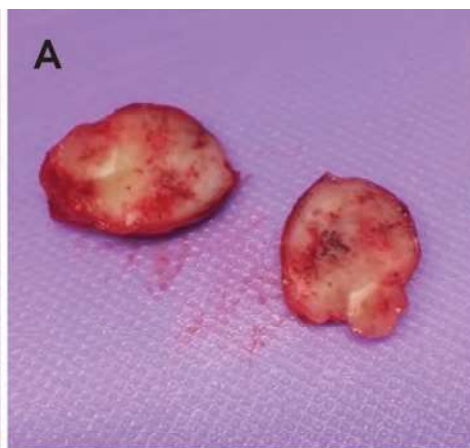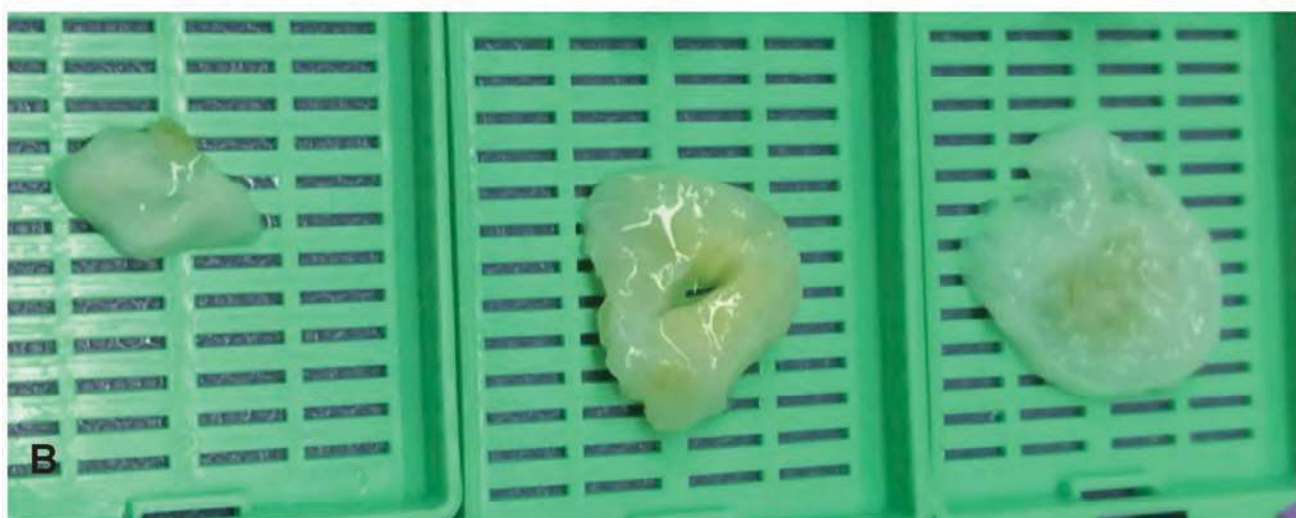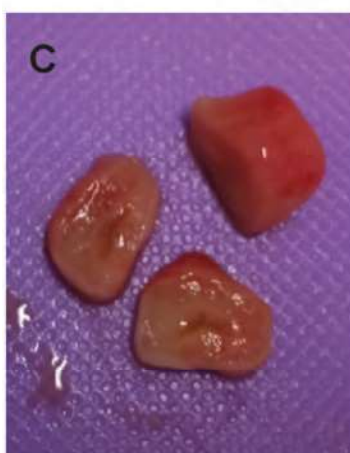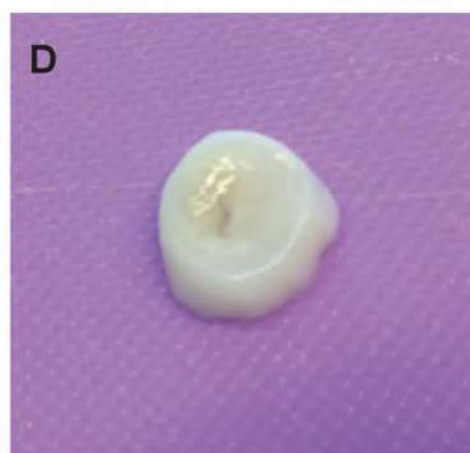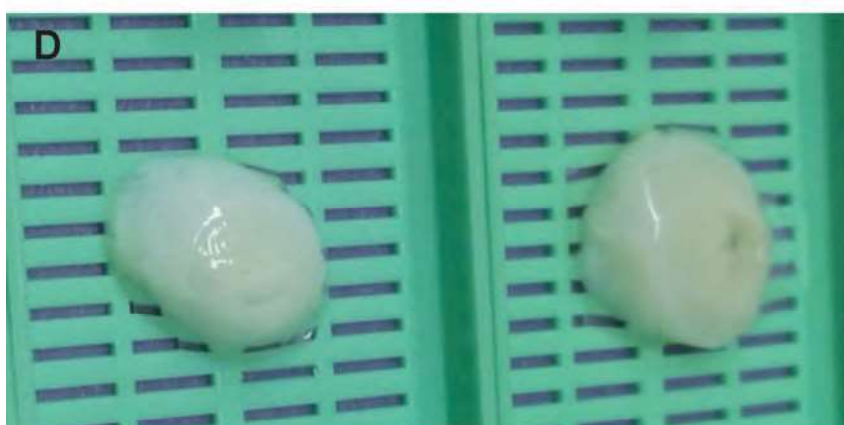

Supplemental figure S1

Supplement: S1 Fig — A) Fibrotic tissue before decellularization. B) Fibrotic tissue after decellularization (SDS). C) Healthy tissue before decellularization. D) Healthy tissue after decellularization (SDS). (PDF) [file pone.0294955.s001.pdf]

Supplemental Figure S2

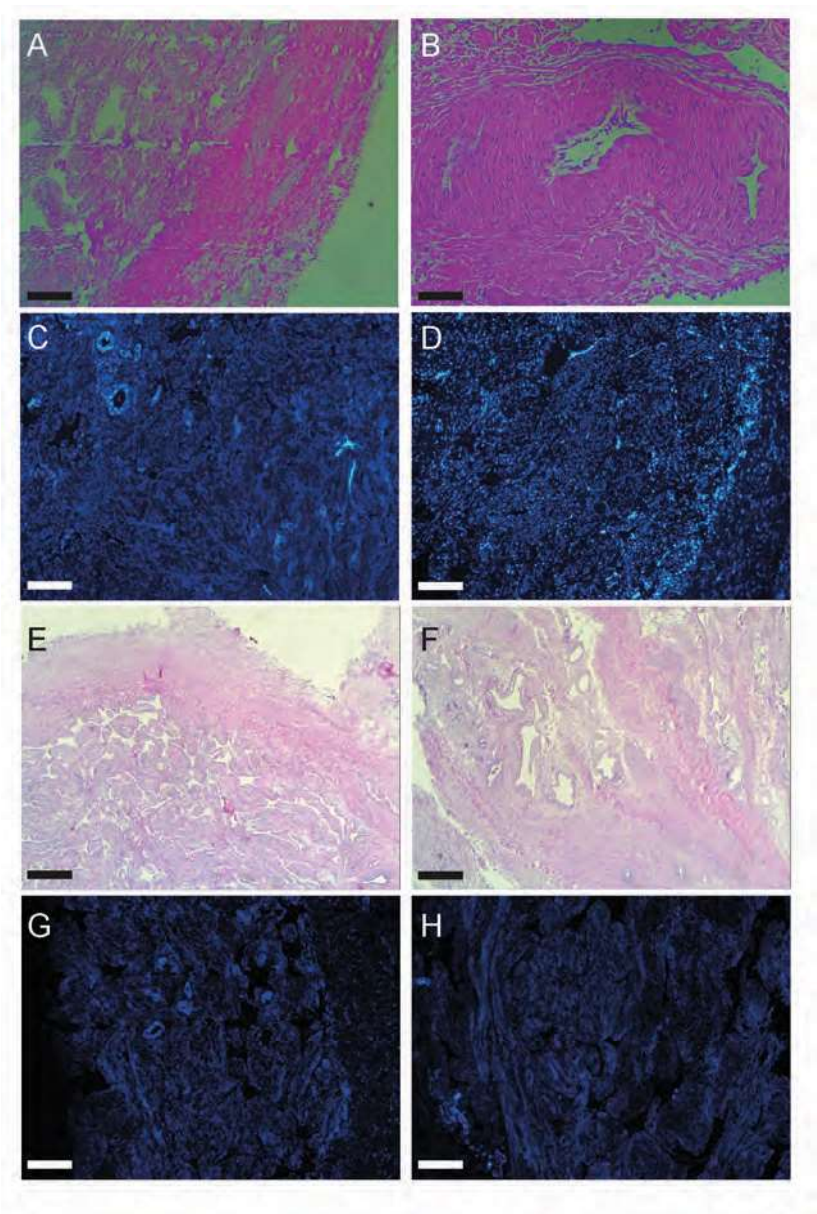

Supplement: S2 Fig — A-D) Triton-X100. E-G) SDS. A, C, E, G, Healthy tissue, B, D, G, H Fibrotic tissue. A, B, E, F: HE-staining. C, D, G, H: DAPI-staining. Scale bar represents 200 μm, except in B and D, scale bar represents 40 μm. (PDF) [file pone.0294955.s002.pdf]

Supplemental Figure S3

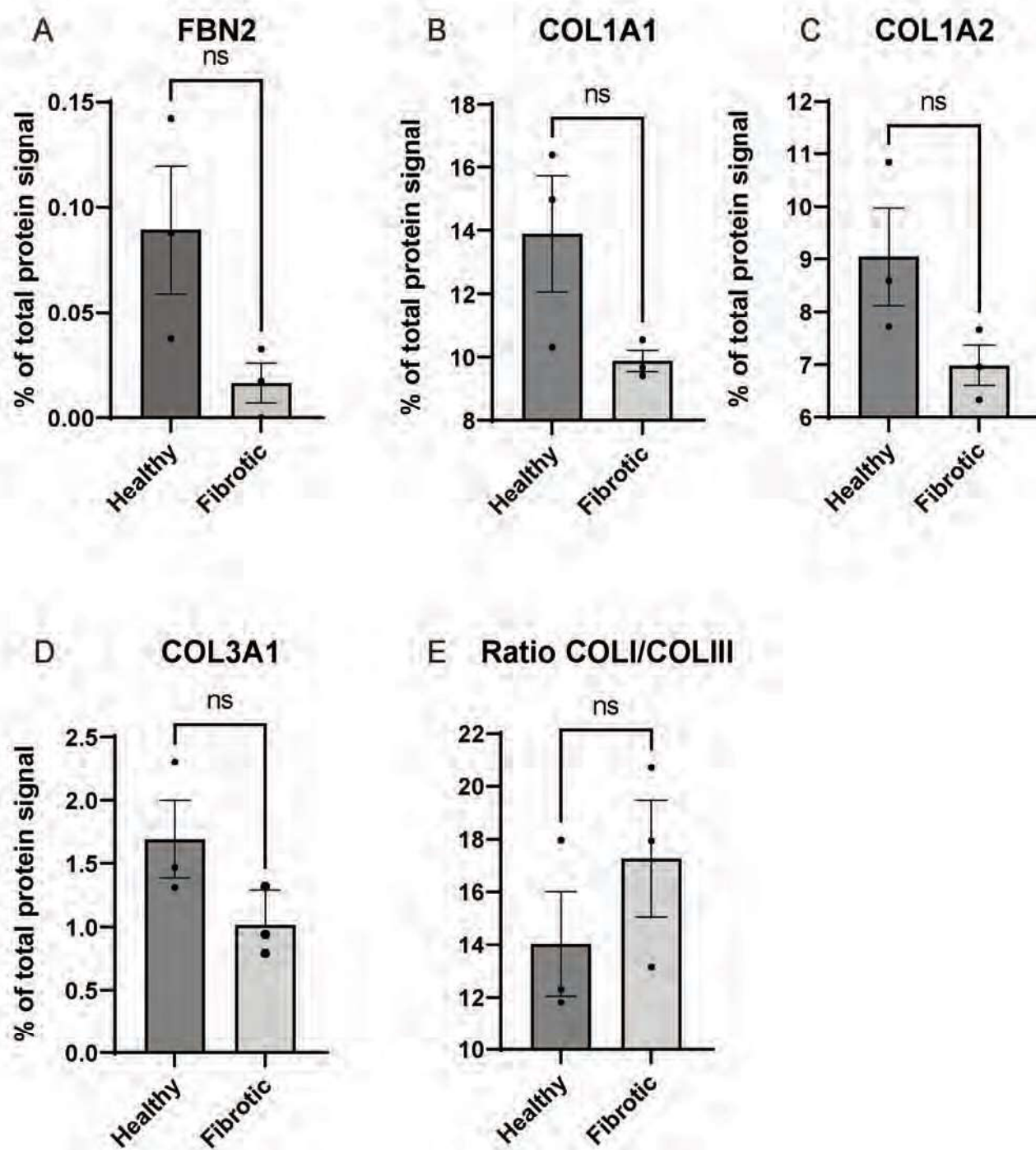

Supplement: S3 Fig — A) FBN2 is downregulated in fibrotic tissue. B) COL1A1 is downregulated in fibrotic tissue C) COL1A2 is downregulated in fibrotic tissue D) COL3A1 is slightly downregulated in fibrotic tissue. For all above panels: Dark grey bars represent healthy tissue, light grey bars fibrotic tissue. Y-axes represent percentage of total protein detected in the decellularized tissue, p≥0.05. D) Ratio COL1/COL3. For this ratio we used (LFQ(COL1A1)+LFQ(COL1A2))/LFQ(COL3A1) per condition. Dark grey bars represent healthy tissue, light grey bars fibrotic tissue. Y-axes represent ratio, although we see a trend of upregulated ratio, the result is not significant (p≥0.05). (PDF) [file pone.0294955.s003.pdf]
