# Supplementary material for: Extracellular matrix analysis of fibrosis: A step towards tissue engineering for urethral stricture disease
Source: PLoS One. 2023 Nov 30;18(11):e0294955. doi: 10.1371/journal.pone.0294955 (PMC10688748; doi:10.1371/journal.pone.0294955)
Supplement: S1 Table — (PDF) [file pone.0294955.s006.pdf]

Table S1. Antibodies used for immunohistochemical stainings.

**Staining**	1st antibody	Dilution 1st antibody	2nd antibody	Dilution 2nd antibody	3rd antibody	Dilution
Collagen I	Mouse anti collagen I; Ab34710 Abcam	1:100	Rabbit anti mouse HRP; Dako P0260	1:100	Poly anti rabbit HRP brightvision, Immunologic	-
Collagen III	Goat anti Collagen type III, SouthernBiotech 1330-01	1:100	Rabbit anti Goat HRP; Dako P0449	1:100	Poly anti rabbit HRP brightvision, Immunologic	-
Collagen IV	Goat anti collagen IV, Merck (Ab769),	1:40	Rabbit anti Goat HRP; Dako P0449	1:100	Poly anti rabbit HRP brightvision, Immunologic	-
Elastin	Mouse anti elastin; Merck mab2503	1:1000	Poly anti rabbit HRP brightvision, Immunologic	-	-	
EMILIN1	Rabbit anti EMILIN1, Sigma atlas antibodies HPA002822	1:100	Poly anti rabbit HRP brightvision, Immunologic	-	-	
FBN1	Rabbit anti FBN1, Sigma atlas antibodies HPA021057	1:100	Poly anti rabbit HRP brightvision, Immunologic	-	-	
