# Supplementary material for: Extracellular matrix analysis of fibrosis: A step towards tissue engineering for urethral stricture disease
Source: PLoS One. 2023 Nov 30;18(11):e0294955. doi: 10.1371/journal.pone.0294955 (PMC10688748; doi:10.1371/journal.pone.0294955)
Supplement: S2 Table — (PDF) [file pone.0294955.s007.pdf]

Table S2: **Matrisome enrichment differs between SDS and Triton decellularization**.

		% of total protein		% of matrisome	
		SDS	TX100	SDS	TX100
Core matrisome	Collagens	14,11	0,00	53,29	0,08
Core matrisome	ECM Glycoproteins	7,18	0,02	27,09	0,66
Core matrisome	Proteoglycans	1,63	0,00	6,17	0,13
Matrisome-associated	ECM Regulators	1,64	0,65	6,19	22,62
Matrisome-associated	ECM-affiliated Proteins	0,66	0,90	2,51	31,36
Matrisome-associated	Secreted Factors	1,26	1,29	4,76	45,15
