# Supplementary material for: Extracellular matrix analysis of fibrosis: A step towards tissue engineering for urethral stricture disease
Source: PLoS One. 2023 Nov 30;18(11):e0294955. doi: 10.1371/journal.pone.0294955 (PMC10688748; doi:10.1371/journal.pone.0294955)
Supplement: S3 Table — (PDF) [file pone.0294955.s008.pdf]

Table S3: **Differential identification of matrisome components**. Proteins in the table were identified in SDS decellularized samples but significantly lower than in the Triton x-100 samples (a >Log2 fold difference). Proteins belonging to the matrisome detected in SDS but not in TX100 are presented below the table

			Log2 difference (SDS vs Triton)
COL1A1	Core matrisome	Collagens	12,18843
COL6A3	Core matrisome	Collagens	11,93907
COL1A2	Core matrisome	Collagens	10,86271
LAMC1	Core matrisome	ECM Glycoproteins	10,21703
FN1	Core matrisome	ECM Glycoproteins	8,533473
TGM2	Matrisome-associated	ECM Regulators	5,216248
VCAN	Core matrisome	Proteoglycans	3,513441

The following proteins of the matrisome were only detected in SDS decellularized and not in TX100 decellularized samples:

COL12A1, COL14A1, COL15A1, COL16A1, COL18A1, COL21A1, COL28A1, COL3A1, COL4A1, COL4A2, COL4A3, COL4A5, COL4A6, COL5A1, COL5A2, COL5A3, COL6A1, COL6A2, COL6A3, COL7A1, COL8A1, COL8A2, AEBP1, CILP2, CTGF, DPT, ECM1, EFEMP1, EFEMP2, ELN, EMILIN1, EMILIN2, EMILIN3, FBLN1, FBLN2, FBLN5, FBN1, FBN2, FGA, FGB, FGG, HMCN2, IGFBP3, IGFBP5, LAMA2, LAMA3, LAMA4, LAMA5, LAMB1, LAMB2, LGI4, LTBP1, LTBP2, LTBP4, MATN2, MFAP4, MFAP5, MFGE8, MGP, MMRN1, MMRN2, NID1, NID2, NPNT, PCOLCE, POSTN, SRPX, THSD4, TINAGL1, TNC, TNXB, VTN, VWA1, VWF, WISP2, AMBP, HPSE2, HRG, HTRA1, ITIH1, ITIH2, ITIH4, ITIH5, LOX, LOXL1, MMP28, SERPINA1, SERPINA3, SERPINA4, SERPINC1, SERPIND1, SERPINF1, SERPINF2, SERPING1, SLPI, SULF1, TIMP3, GREM1, ASPN, BGN, DCN, HSPG2, LUM, OGN, ANGPTL2, CHRDL1, CXCL12, MDK, SCUBE3, TGFB1I1, TGFBI, TNFSF13;TNFSF12-TNFSF13, WNT2B, WNT5A, WNT9A
