# Supplementary material for: Extracellular matrix analysis of fibrosis: A step towards tissue engineering for urethral stricture disease
Source: PLoS One. 2023 Nov 30;18(11):e0294955. doi: 10.1371/journal.pone.0294955 (PMC10688748; doi:10.1371/journal.pone.0294955)
Supplement: S4 Table — (PDF) [file pone.0294955.s009.pdf]

Table S4: Fold change of different matrisome components as depicted in the Vulcano plot of Figure 4C. In green the collagens, in red the glycoproteins, yellow the proteoglycans, in purple the ECM regulators, in blue the ECM affiliated proteins and lastly in orange the secreted factors. Fold change is significant with a p-value < 0.05 (indicated by *)

**Collagens**		
	Fold Change	P value
COL1A1	0,711377	0,098229
COL1A2	0,772185	0,109246
COL3A1	0,600564	0,121841
COL4A1	0,936854	0,455015
COL4A2	1,234005	0,03524*
COL4A5	0,816778	0,212837
COL4A6	0,951064	0,794649
COL5A1	0,643508	0,213933
COL5A2	1,077303	0,682598
COL6A1	1,180478	0,542117
COL6A2	1,106956	0,68526
COL6A3	1,430824	0,426415
COL8A1	1,466713	0,435661
COL8A2	0,936117	0,930986
COL12A1	1,807232	0,155444
COL14A1	1,068128	0,745797
COL15A1	1,66403	0,014922*
COL16A1	0,545585	0,202226
COL18A1	0,862431	0,491079
COL21A1	0,596638	0,059649
COL28A1	4,325162	0,053244

**Glycoproteins**		
	Fold Change	P value
ABI3BP	1,055261	0,912952
AEBP1	3,376402	0,095955
CILP	37,43565	0,156786
CILP2	4,914764	0,427881
COMP	3,112187	0,185357
DPT	1,233365	0,202797
ECM1	1,438975	0,645729
EFEMP1	0,771475	0,426284
EFEMP2	2,17567	0,47822
ELN	0,758776	0,579569
EMILIN1	1,272193	0,217424
EMILIN2	0,487316	0,028975*
EMILIN3	2,694016	0,059978
FBLN1	0,740031	0,325996

FBLN2	0,505518	0,215657
FBLN5	0,744599	0,500445
FBN1	1,012512	0,944331
FBN2	0,187827	0,084261
FGA	2,96594	0,19573
FGB	5,839732	0,224696
FGG	4,363258	0,212556
FN1	1,759911	0,315868
HMCN2	1,070096	0,937154
IGFBP5	0,477676	0,241195
LAMA2	0,844021	0,765398
LAMA3	1,6514	0,295869
LAMA4	1,669475	0,212744
LAMA5	1,428619	0,29347
LAMB1	1,514358	0,321782
LAMB2	1,520486	0,255264
LAMC1	1,43677	0,327164
LGI4	0,497146	0,242334
LTBP1	0,575393	0,08969
LTBP2	1,30299	0,440756
LTBP4	1,056221	0,877224
MATN2	0,802374	0,630257
MFAP2	0,930403	0,698173
MFAP4	0,848237	0,786142
MFAP5	0,699481	0,026*
MFGE8	4,464442	0,235947
MGP	0,651044	0,784491
MMRN2	2,100047	0,660909
NID1	1,170226	0,564491
NID2	1,591437	0,228267
NPNT	1,136973	0,290577
NTN1	0,667995	0,796167
POSTN	0,346793	0,004365*
SBSPON	2,26315	0,53581
SRPX	1,107445	0,854977
SRPX2	3,254186	0,119837
TGFBI	1,504507	0,158299
THSD4	1,00746	0,981012
TINAGL1	1,518673	0,496497
TNXB	4,471837	0,343845
VTN	0,793825	0,63655
VWA1	2,552144	0,244404
WISP2	2,033985	0,363017

Proteoglycans		
	Fold Change	P value
ASPN	1,021316	0,968312
BGN	1,091451	0,740243
DCN	1,067674	0,73529
HSPG2	1,007367	0,942417
LUM	0,952035	0,596717
OGN	1,552765	0,450015
PODN	1,195318	0,736783
PRELP	0,976252	0,915393
PRG2	0,641512	0,77789
VCAN	0,439595	0,153861

ECM regulators		
	Fold Change	P-value
A2M	2,097404	0,184514
ADAMTS4	0,358673	0,349507
ADAMTS5	0,47973	0,433748
ADAMTSL1	1,065081	0,959737
AMBP	1,160463	0,584852
CSTA	0,679198	0,260101
F9	0,7687	0,748312
HPSE2	1,02442	0,982016
HRG	1,822977	0,145963
HTRA1	1,601288	0,25666
ITIH1	0,750334	0,568379
ITIH2	2,50674	0,466003
ITIH3	2,067322	0,499274
KNG1	1,812661	0,23935
LOX	1,159775	0,802352
LOXL1	0,725585	0,318488
MMP23A	0,46305	0,397339
MMP28	0,56355	0,214804
PLG	1,398593	0,5165
SERPINA1	0,665613	0,144988
SERPINA3	1,014807	0,947519
SERPINA4	3,309852	0,09024
SERPINA5	0,68432	0,807325
SERPINC1	0,970847	0,853168
SERPINE2	0,472204	0,361904
SERPINF2	3,13939	0,06594
SERPING1	0,635069	0,773413
SLPI	0,622263	0,525625
SULF1	1,558044	0,386778

TGM2	1,943741	0,0593
TIMP3	0,569131	0,415693

affiliated factors		
	Fold Change	P value
ANXA1	2,076215	0,343762
ANXA2	1,363073	0,227564
ANXA5	1,469602	0,458936
ANXA6	1,927511	0,16475
ANXA7	2,824441	0,360258
C1QB	0,923196	0,892853
C1QBP	1,081059	0,958744
C1QC	2,924545	0,160638
C1QTNF5	1,015272	0,991963
GREM1	0,388132	0,362747
HPX	2,886029	0,344678
LGALS1	0,873717	0,654163
LGALS3	2,765437	0,154903
SEMA3B	1,232786	0,727448

secreted factors		
	Fold Change	P value
ANGPTL2	1,036139	0,933304
ANGPTL4	1,516584	0,722763
CHRDL1	3,490203	0,127817
CXCL12	0,843432	0,645474
FGF7	2,09269	0,413196
HRNR	0,672474	0,229201
IL17D	0,583164	0,22388
MEST	0,500428	0,147456
PDGFB	1,121279	0,921285
S100A11	1,397191	0,718688
S100A4	0,948278	0,855038
S100A6	0,689901	0,109249
S100A7	0,514982	0,244955
S100A8	0,466804	0,303282
S100A9	1,191074	0,647083
SCUBE3	1,039615	0,948466
WNT16	0,694335	0,787571
WNT2	0,705766	0,821844
WNT2B	0,858232	0,776125
WNT5A	0,312495	0,274822
WNT5B	1,481893	0,800837
WNT6	0,972917	0,962752

WNT9A	2,30872	0,269343
