# Supplementary material for: Extracellular matrix analysis of fibrosis: A step towards tissue engineering for urethral stricture disease
Source: PLoS One. 2023 Nov 30;18(11):e0294955. doi: 10.1371/journal.pone.0294955 (PMC10688748; doi:10.1371/journal.pone.0294955)
Supplement: Attachment — Submitted filename: response to reviewers PLOS ONE.docx [file pone.0294955.s010.docx]

Response to reviewers on manuscript PONE-D-23-17947: Extracellular matrix analysis of fibrosis: a step towards tissue engineering for urethral stricture disease

First, we would like to thank the editor and the reviewers for their time and careful assessment of the manuscript. Below you will find a point-by-point reply to the concerns in the manuscript. The tracked changes in the manuscript can be found in the separate uploaded document.

Reviewer #1:

1. In the current routine urethral reconstruction, urethra is only reconstructed with epithelial tissue, and the corpus spongiosum (CS) is not reconstructed. Literature on CS reconstruction is scarce. The author aims at the reconstruction of CS, which should be appreciated.

*We appreciate the time and dedication the reviewer spent on the manuscript. This is exactly the message we meant to address in our research group, to improve urethral reconstruction by including the CS. This study is a step forward in the knowledge to achieve this.*

2. The reconstruction using tissue engineering technique should be based on normal cells, normal structure and normal extracellular matrix of normal healthy tissue in order to fulfill the biomimetic reconstruction. Moreover, In real surgery, In real urethral stricture disease, there is fibrosis of the urethral epithelium and deeper layers including the CS. Urethral strictures can be best treated with open urethroplasty, where the fibrotic urethra and CS is completely excised, followed by primary anastomosis on the site of healthy tissue could be found. Therefore, if tissue engineering reconstruction of urethra is the goal of this research, the focus of research should be on the normal urethra, normal epithelia, normal CS and its normal extracellular matrix. The present study focused on the fibrotic CS and its extracellular matrix within the urethral stenosis, The information provided to us should primarily facilitate the comprehension of corpus spongiosum fibrosis formation mechanism, while being restricted for tissue engineering research on CS.

*Thank you for this comment. We completely agree with the reviewer that the ultimate goal is to create healthy CS for the purpose of urethral stricture treatment. In this study we compared fibrotic and healthy CS. Knowledge of the ECM of fibrotic CS is essential in order to be able to inhibit certain proteins to prevent fibrosis.*

3. The author focuses on the proteomic comparison of normal spongiosum and fibrotic spongiosum in urethral stenosis, maybe he wants to provide strategies for the development of tissue engineering spongiosum to avoid the fibrosis formation after they are transplanted into body. If so, the fibrosis of tissue engineered constructs is mainly due to insufficient blood supply of the construct, lack of vascularization leads to fibrosis and graft rejection, which is completely different from the fibrosis of spongiosum caused by urethral stenosis. Furthermore, the authors' proteomic study of the extracellular matrix of urethral spongiosum only presents findings without exploring the specific roles of these altered proteins in urethral stricture and CS fibrosis. As a result, their work merely offers a comparison of phenomena rather than an investigation into underlying mechanisms of urethral stricture or potential applications for tissue engineering CS.

*We agree with the reviewer that the fibrotic phenomena in tissue engineering may be different from spongiofibrosis. As the CS is a vascular structure, in our opinion, tissue engineered CS should actually prevent fibrosis due to insufficient blood supply. However, also after transplantation of a tissue engineered CS and endothelium there is a lurking danger of fibrosis caused by urine leakage. That is why we think that detailed data on protein composition of fibrotic CS could contribute to understanding disease and healthy healing.*

Reviewer #2:

The authors presented a proteomic analysis of healthy vs diseased corpus spongiosum using LC-MS/MS. The corpus spongiosum is an important component of the male urethra which provides copious vascular supply and physical protection. It is interesting that the distribution of protein types were similar between each groups.

The largest limitation is the unclear history of both "healthy" controls, who are likely to have had hormonal exposure and unclear history of disease controls (lichen sclerosis v injury v prior urologic procedure v first stricture repair v hx of hypospadias). If known, please discuss the background of diseased specimens further.

*All patients included are biobank patients where we collect waste material, which means we don’t have full access to all medical records. What we know is the diseased fibrotic tissue originated from the bulbar region, after an excision and primary anastomosis procedure. Therefore, hypospadias or lichen is not expected, as strictures with these origins occur more in the penile region. We have included the sentence; “*All patients had a history of at least one endo-urethral treatment (direct vision internal urethrotomy or endoscopic dilatation). The cause of the primary stricture (traumatic, inflammatory, iatrogenic) was unknown.”*(line 77-79)*

*The ‘healthy’ tissue after gender conformation surgery originates from the penile urethra. Patients were treated with feminizing hormones (see also de Graaf et al, 2018 PMID: 29985521), however, we did not have access to full medical records. As it is very difficult to obtain healthy human urethral tissue, in our opinion, this was the best alternative.*

Discussion mentioned the limitation of using the transgender population as healthy controls, but should authors should discuss how hormones are known to effect corpus spongiosum (i.e. in hypospadiac infants) and if there is literature on hormone changes on the proteins that were up/downregulated.

*The influence of hormones on ECM has already been addressed in the discussion:* It is not known how these altered hormone levels influence the CS, but there is evidence that hormones can affect ECM proteins (32, 33). *The action of (altered) hormones and hypospadias may be very different from influence of feminizing therapy in transgender patients, so this* *topic will not be included in the discussion.*

Title:
--It would be helpful to include the methods (i.e. proteomics via LC-MS/MS) in the title

*We thank the reviewer for this suggestion, however, as we also use histochemistry and immunohistochemistry we believe that ECM analysis covers both methods.*

Abstract:

--clear, concise, interesting

Introduction:

--well written, no suggestions

Methods:

--Diseased tissues, did these patients have lichen sclerosis, injury, prior urologic procedure, prior repair or were these all first presenting urethral stricture disease?

*We have discussed this now in the M&M, see line 77-79*

--Control tissues were obtained from gender confirmation surgery. Limitations of this are briefly mentioned in the discussion.

--How was an n-number of 3 chosen?

*Due to limitation by scarcity of the normal tissue we chose to have equal groups in the analysis, therefore 3 of each.*

Results:
--Tables/figures/graphs are clear, concise, with appropriate labels and error bars

Discussion:
--Good discussion of up and downregulated proteins in fibrotic tissues. Could discuss how hormone therapy may effect these proteins in particular to provide more rationale for why transgender tissue is sufficient in this analysis.

*We have added a part in the discussion on this, see page line284-287 and explanation*	*above.*

Reviewer #3:

Authors study the extracellular matrix of the fibrous tissue in regions of excised penile urethral strictures and compare these to normal extracellular matrix from corpus spongiosum taken from transgender procedures. The authors demonstrate differences in subtypes of extracellular matrix materials between fibrotic and normal samples, which may aide augmenting urethral reconstructive surgery which currently only involves mucosal grafting. Authors postulate that deficient biomatrix materials may be bioengineered as a future aim for surgical improvement. While the study is limited in the number of samples (3 strictured and 3 “normal”) the analysis is well done and nicely validated. The discussion is excellent with well described function of deficient matrix proteins in other organ fibrotic conditions and a very nicely written paragraph discussing the limitations.

*We like to thank the reviewer for the dedicated time spent on the manuscript. Below we answer the major and minor issues on the manuscript, together with referral to the page/line in the manuscript itself.*

Major:
The tables cited in the text are not provided in the manuscript download. Supplemental tables are presented but not the primary Tables which would need to be reviewed in context with the manuscript.

*We apologize for this error, we have included the tables in the revised version.*

Minor:

1. Methods: Please state in decellularization procedure whether you are working with frozen tissue or FFPE. I assume the former but it should be stated.

*We have added ‘frozen’ in the materials and methods on page 6*

2. Results (line 191): inter-alfa-trypsin should be inter-alpha-trypsin

*Thank you, this is corrected*

3. Results –“validation of ECM in Immunohistochemistry section”: It isn’t clear why you stained FBN1 and EMILIN1 when your results show differences in FBN2 and EMILIN2. I assume it is because of antibody availability for 1’s and not 2’s. Could you please state why you took this approach.

*Indeed, this is partly because of antibody availability, in addition also the expression levels of the FBN1 and EMILIN1 are 40-700 fold higher than of the 2-isoforms (see table 4). As the isoforms are similar in function and in interactions, we choose to analyse the 1-isoforms. We have now added a part in the discussion (line 289-292) on this.*

4. Discussion (line 232, 243, 251): “higher expressed” consider rewording “more highly expressed”

*Thank you for this suggestion, we rephrased this.*

5. Discussion (lines 243-253): In the discussion of POSTN – nice discussion of findings related to skin wound healing. You mention that POSTN is produced in pericytes. One difference between CS and skin is that the erectile tissue is rich in pericytes in the normal state. Some of the difference you describe may relate to loss of the pericytes in the process of “spongiofibrosis”.

*Thank you for this suggestion, this is very well possible. It is known from literature there is a function for pericytes in erectile function (PMID: 26044953). We have added this in the discussion (line 256-258)*

Reviewer #4:

Despite the burgeoning numbers of papers related to interstitial fibrosis of various organs during the past decade, many papers under report on the actual composition of the ECM scaffold in the fibrosing tissues. If in some cases, the changes in the nature and composition of the ECM matrix is reported, it may reflect a cursory investigation of relative mRNA abundance of the usual fibrillar collagens, including type I and type III alpha-monomers. While the matrix is, in some organs, dominated by these two fibrillar collagen species, the ECM is complex and a brief survey too often diminishes the role(s) of other protein components of the altered ECM. In the current paper, the authors sought to develop a tissue engineering strategy for alleviating issues found with the fibrosed urogenital tract, and point out that “it is necessary to know the protein composition of the corpus spongiosum”. While true, this approach requires the development of some innovative methodologies, or at least, an extension of those techniques. In the current work the authors analyzed the localization of ECM components in human healthy and fibrotic corpus spongiosum using immunohistological techniques. The overarching experimental design included an assessment of healthy normal tissues as well as fibrosed samples. In general the paper is very well written and the techniques used were adequate – further, the conclusions were within the confines of the data presented. The impact of the work could be improved if the following comments are given some attention:

Specific comments

1. Is it possible to include additional results eg, IF staining for collagen 4 alpha 1 within Figure 1? This would inject a modicum of novelty to the depiction of collagens I and III.

*We have added a collagen4 staining, figure 2 E and F.*

2. Matrisome proteins depicted in the volcano plot of Figure 3 is one of the best Figures of the paper, and provides some specific and interesting data. Again it appears to represent the tip of the iceberg so to speak. Is it possible to select two or three proteins from within each generalized group of matrisome protein classes to highlight these results in a separate panel? The ECM associated proteins and “regulators” are not well represented in the current literature.

*This information is given in Table 3 for specific proteins mentioned in the text. We have added a table of all proteins depicted in the volcano plot now in supplemental table S4*.

3. Figure 5 shows elastin staining. Elastin is notoriously slow to degrade and is almost never added to tissues. Alternatively, elastin fenestration is a dynamic process that is ongoing with ageing tissues and may be altered in fibrosis. Is there any data to interrogate the status of elastase activities in CS samples (healthy and fibrosed)? Please comment.

*We fully agree on the complications in elastin analysis, whereas in the proteomic analysis we did not find a difference in amount of peptides, we found a difference in the distribution in the tissues. Also, in the proteomic analysis we found many different hits for elastin, however, after careful analysis none of these isoforms were significant. Unfortunately, we have no data on elastase activity in the tissue. While interesting, we cannot perform this as we do not have tissue where enzymatic activity is preserved. Collecting new tissue would give a new variable in the data we already have.*
